# Will we ever wash our hands of lubrication theory?

**DOI:** 10.1063/5.0060307

**Published:** 2021-08-17

**Authors:** Paul S. Hammond

**Affiliations:** Hammond Consulting Limited, 62 High Street, Bourn, Cambridge CB23 2TR, United Kingdom

## Abstract

Lubrication theory is used to investigate how weakly bound particles can be transported away from the vicinity of the wall when two spatially periodic rough surfaces are sheared relative to one another at constant velocity *U* while immersed in fluid. The aim is to model what could be an important process during decontamination of hands by washing and is motivated by Mittal *et al.* [“The flow physics of COVID-19,” J. Fluid Mech. **894**, F2 (2020)] who remark “Amazingly, despite the 170+ year history of hand washing in medical hygiene, we were unable to find a single published research article on the flow physics of hand washing.” Under the assumption that the roughness wavelength 2π/k is large compared with the spacing of the surfaces, *a*, the lubrication approximation permits closed-form expressions to be found for the time-varying velocity components. These are used to track the motion of a particle that is initially trapped in a potential well close to one of the surfaces, and experiences a drag force proportional to the difference between its velocity and that of the surrounding fluid. Complications such as particle-wall hydrodynamic interactions, finite size effects, and Brownian motion are ignored for now. Unsurprisingly, particles remain trapped unless the flow driven by the wall motion is strong compared to the depth of the trapping potential well. Perhaps less obvious is that for many starting positions the process of escape to large distances from the wall takes place over a large number of periods 2π/kU, essentially because the no-slip boundary condition means that fluid velocities relative to the wall are small close to the wall, and thus the velocities of particles along or away from the wall are also small. With reasonable estimates for the various dimensional parameters, the escape times in these cases are found to be comparable in magnitude to the washing times recommended in hand washing guidelines.

## I. INTRODUCTION

Few would doubt that good hand hygiene can help control the spread of disease and minimize risks of infection, even if airborne transmission may be more important in the COVID-19 context.^2–5^ So it is surprising to read that the physical processes involved in hand washing have, apparently, not been studied by fluid mechanicians; see Sec. IV C of Mittal *et al.*^1^ Maybe the very familiarity, simplicity, and technology-independence of the act of washing ones hands means that it goes unnoticed and unquestioned outside specialist health and development communities, or perhaps the issue is the very lack of prior literature, in which case this paper, which puts forward a simple fluid mechanical model for a process of particle removal during hand washing, might provide a starting point.

Guidelines on good hand washing practices can be found in WHO^6^ and a collection of videos of hand washing in Lulla *et al.*^7^ It is clear from the WHO guidelines that thoroughness is necessary to achieve good results (the guidelines detail a seven step sequence to ensure all parts of the hand are addressed), and that time and relative motion are important (the hands are rubbed together throughout, and the guidelines imply that around 5 s should be spent on each step). A quick splash under the tap is certainly not enough, which suggests that dislodging or deactivating unwanted material is not trivially easy. The remainder of this paper sets out a fluid mechanical model for particle removal that explains why this is the case.

More information is available in the literature about the chemical processes involved in the deactivation of bacteria and viruses, perhaps because cleaning products are the foundation of multi-billion dollar global businesses, giving a commercial incentive for understanding-based innovation. See Kampf and Kramer^8^ for a review of pathogens and control agents, Golin *et al.*^9^ and Singh *et al.*^10^ for information on formulation of alcohol-based hand sanitizer and its mode of action on coronaviruses, and Poon *et al.*^11^ for a COVID-focused survey of the soft matter science involved. In very broad terms, it seems that suitable molecules, usually amphiphilic, are able to incorporate themselves into the membranes surrounding a bacterium or virus, leading to disruption of the membrane or its function, and thence destroying the pathogen or damaging it sufficiently that it is no longer a threat. Given this picture, the key fluid mechanical process is getting the chemical agent to the location of the pathogen, i.e., transport of an externally supplied molecule to a target (initially) on the surface of the hand. The fluid mechanics of convection-diffusion taking place in the flow occurring in the inter-hand region during washing is thus highly relevant.

It is unlikely that the target referred to in the previous paragraph is, in the COVID-19 context, a simple unadorned virus particle. More likely it is a mixed entity resulting from the aging of a deposited exhaled droplet containing viruses, water, salts, lipids, and biopolymers,^11–13^ with further complications arising from re-wetting when the hand washing medium is applied. This adds a significant uncertainty to the quantification of the target size and the magnitude of the forces holding it in place.

In the light of the previous remarks, we study the flow in the fluid-filled gap between the surfaces of relatively moving hands, the transport of chemical species in this gap, and the processes whereby a particle initially on one of the hand surfaces is removed into the bulk fluid. Since there is little prior fluid-mechanical literature, it is reasonable to make a start by employing a simple mathematical model. Complications can be added later as the need is revealed from consideration of the results of these initial investigations. The lack of quantitative data on various parameters, such as the strength of virus-surface interaction forces, is another reason to avoid excessive complexity and to try to identify features that are not sensitively dependent on parameter values.

Hence, we shall here study flow and transport in a simple geometry, chosen so that the fluid flow problem can be solved analytically. The hands are modeled as two rough walls, closely spaced to one another, with roughness wavelengths long compared to the width of the fluid-filled gap. This allows the fluid flow in the gap to be calculated using the time-honored lubrication approximation, permitting simple closed-form expressions to be given for the velocity components. This velocity field is then used to track the trajectories of particles taking account of short-range forces between particle and hand and assuming the simplest possible slip-related drag form for the hydrodynamic interaction between flow and particle. Particles are taken to be attracted to the wall by a short-range force and to be repelled at very close approach so as to avoid interpenetration, hence, in the absence of flow they would be trapped in a potential well close to the wall.

Despite the simplicity, this model exhibits some interesting features, for example, the existence of a threshold relative velocity between the hands below which particles are not removed from the potential well binding them to the wall, and allows these features to be explained in a quantitative fashion. Also, the model explains why removal of particles from the wall region requires a significant time, when measured in units of 2π/kU which is the time for the surfaces to translate by one roughness wavelength, thus perhaps providing a mechanistic justification for the recommendations in the guidelines on the time to be spent during hand washing. The long time required is a direct consequence of the smallness of fluid–solid relative velocities near the wall, which in turn is a consequence of the no-slip boundary condition.^14^

It might be objected that direct solid–solid mechanical interactions at points of close approach of the surfaces may play a role in particle removal, and this possibility is not considered here. This is true, but since not every point on a pair of rough surfaces can experience direct interaction, unless the surfaces are so deformable that they become conformant, the present purely hydrodynamic model must have some relevance even if it is not complete.

The remainder of this paper is organized as follows: Sec. II details the study geometry, and Reynolds and Péclet numbers are evaluated to identify the controlling process dynamics. The equations governing fluid flow are given in Sec. III, and solved under the approximation that gap width is small compared to the length scale of axial variations. The forces on a virus particle and the equation governing its motion under the combination of flow and wall-attraction forces are specified in Sec. IV, and a dimensionless group measuring the relative importance of wall attractions to flow forces is derived. Particle trajectories are computed and displayed, in Sec. V, and it is found that particles are not removed from the wall region unless flow forces are strong enough to overcome the wall attraction. An order of magnitude explanation is given for the critical value of the dimensionless parameter, and the timescale of particle removal. Finally, key conclusions and areas where the model might usefully be extended or revised are discussed in Sec. VI.

Despite its age^15^ it seems that the lubrication approximation still has something useful to give in new contexts, and so is likely to be with us for some time yet.

## II. GEOMETRY AND BASIC PARAMETERS

The hands are modeled as two rough surfaces in relative motion separated by a film of fluid. The situation is taken to be two dimensional, with *x* being the coordinate in the axial direction and *y* in the transverse. The upper surface is located at $y=h_{+}(x,t)$, the lower at $y=h_{-}(x,t)$. In order to simplify the velocity calculation we shall demand both symmetry about *x* = 0 such that $h_{-}(x,t)=-h_{+}(-x,t)$ and also periodicity such that $h_{+}(x+(2\pi /k),t)=h_{+}(x,t)$ for all *x* (and similarly for $h_{-}(x,t)$ of course). Simple forms are used here,

$$h_{\pm }(x,t)=a[\pm (1+\frac{\eta }{2})+\text{sin}\;k(x\mp \frac{Ut}{2})].$$ (1)

It will be assumed throughout that axial roughness wavelength scale, 2π/k, is much greater than the transverse gap width length scale, *a*, so that the parameter ka≪1. The relative velocity of the walls in the *x* direction, *U*, is taken to be constant in time.

The relative importance of advection of suspended or dissolved material relative to diffusion is measured by the Péclet number Pe=aU/D, where *D* is the particle or molecular diffusivity. For modest sized surfactants $D=O(10^{-9})\;m^{2}/s$, while D=kT/6πdμ for a solid particle of radius *d*, so when d=O(50 nm), characteristic of a virus particle,^11^ T=300 K and the suspending fluid viscosity $\mu =10^{-3}\;\text{Pa}\;s$ then $D=4\times 10^{-11}\;m^{2}/s$. Taking $U=O(10^{-1})\;m/s$, and $a=O(10^{-5}\;m)$ which is shown in Sec. III B to be reasonable, then Pe lies in the range 10^3^ to $2\times 10^{4}$. In the geometry considered, for both surfactants and virus sized particles, diffusion effects are therefore weak compared with advection. At least for a first investigation, simple non-Brownian particle tracking should yield some useful insights. The ratio between the virus diameter and the film thickness, for the parameters given, is $2d/a=10^{-2}$, introducing another small geometrical parameter into the problem.

The Reynolds number is Re=aU/ν, where *ν* is the kinematic viscosity of the fluid filling the gap between the walls, and for the parameters given above Re=O(1). When ka≪1 the appropriate measure of inertia effects is the modified Reynolds number^16^ kaRe, which will be small provided $ka<10^{-1}$ (and the same factor acts on Pe in lubrication geometry). Smaller values for *a* will reduce Re further. Neglect of inertia is probably justifiable in a first-look context, but maybe not beyond, and certainly not if axial length scales are comparable with film thicknesses. Much depends on the value assumed for *a*, which is dictated by a combination of surface roughness and the interaction of applied loads and surface deformability, and as such is hard to estimate accurately. Turbulence, which would significantly enhance mixing, can probably be ruled out, however, since at least a three order of magnitude increase in *a* would be required, taking it to a value rather large for hand washing, but convective chaos resulting from geometrical irregularity and changes in *U* remains a possibility even at low Reynolds number.

Writing *u_slip_* for the magnitude of the difference between the particle and fluid velocities, the particle slip and shear Reynolds numbers are $Re_{\mathrm{slip}}=du_{\mathrm{slip}}/\nu$ and $Re_{\mathrm{shear}}=d^{2}U/a\nu$ and are smaller than the bulk Reynolds number Re by factors of $\left(d/a)(u_{\mathrm{slip}}/U\right)$ and ${\left(d/a\right)}^{2}$, respectively. Since $u_{\mathrm{slip}}/U\leq 1$, and $d/a=O(5\times 10^{-3}),\;Re_{\mathrm{slip}}\leq O(5\times 10^{-3})$, and $Re_{\mathrm{shear}}=O(2.5\times 10^{-5})$. This implies that forces arising from fluid inertia have a negligible effect on particle motion when compared to drag.^17^

Where appropriate dimensionless variables, denoted with a hat, will be used in the sequel. Lengths will be scaled with *a*, velocities with *U*, and times with *a*/*U* so that $\hat{x}=x/a$, etc.

## III. FLUID FLOW

### A. Governing equations and boundary conditions

Under the assumption that fluid inertia effects are negligible, the fluid velocity u=(u,v) and pressure *p* satisfy

$$\nabla \cdot u=0,\;\mu \nabla^{2}u=\nabla p,$$ (2)

where *μ* is the fluid viscosity. Making the lubrication approximation Eq. (2) becomes

$$\frac{\partial v}{\partial y}=-\frac{\partial u}{\partial x},\;\mu \frac{\partial^{2}u}{\partial y^{2}}=G(x,t),$$ (3)

and is subject to boundary conditions

$$u(x,h_{\pm },t)=\pm \frac{U}{2},\;v(x,h_{\pm },t)=0.$$ (4)

The axial volume flux *Q* at any axial position, *x*, is

$$Q(x,t)=\int_{h_{-}}^{h_{+}}u(x,y\prime ,t)dy\prime ,$$ (5)

and differentiating this expression and using 3(a) and 4(b) we find

$$\frac{\partial Q}{\partial x}=\frac{U}{2}(\frac{\partial h_{+}}{\partial x}+\frac{\partial h_{-}}{\partial x}).$$ (6)

Then, integrating with respect to *x*, and fixing the constant of integration by observing that Q(0,t)=0 as a result of symmetry together with the requirement that there is no injection or removal of fluid as x→±∞, it follows that

$$Q=\frac{U}{2}(\Delta h_{+}+\Delta h_{-}),$$ (7)

where $\Delta h_{\pm }=h_{\pm }(x,t)-h_{\pm }(0,t)$.

### B. Velocity field and pressure gradient

Equation (3) is easily integrated to obtain

$$u=U\frac{y-\frac{h_{+}+h_{-}}{2}}{h_{+}-h_{-}}+\frac{G}{2\mu }(y-h_{+})(y-h_{-})$$ (8)

$$=U[A(x,t)y^{2}+B(x,t)y+C(x,t)].$$ (9)

Upon integrating Eq. (8), we find that

$$Q=-\frac{G}{12\mu }{\left(h_{+}-h_{-}\right)}^{3}.$$ (10)

Equating this expression to Eq. (7), it follows that the pressure gradient is

$$G=-6\mu U\frac{\Delta h_{+}+\Delta h_{-}}{{\left(h_{+}-h_{-}\right)}^{3}}.$$ (11)

The tangential shear stress exerted on the lower wall by the fluid is

$$\mu \frac{\partial u}{\partial y}(x,h_{-})=\mu U\left[\frac{1}{h_{+}-h_{-}}+3\frac{\Delta h_{+}+\Delta h_{-}}{{\left(h_{+}-h_{-}\right)}^{2}}\right],$$

the first term coming from the linear shear flow and the second from the parabolic pressure-driven contribution. On the upper wall, the same terms appear but the sign of the first contribution is reversed. The order of magnitude of wall shear stresses is O(μU/a). Using the expression for *G* Eqs. (8) and (9) implies

$$A=-3\frac{\Delta h_{+}+\Delta h_{-}}{{\left(h_{+}-h_{-}\right)}^{3}},$$

$$B=\frac{1}{h_{+}-h_{-}}+3\frac{\Delta h_{+}+\Delta h_{-}}{{\left(h_{+}-h_{-}\right)}^{3}}(h_{+}+h_{-}),$$

$$C=-\frac{h_{+}+h_{-}}{2(h_{+}-h_{-})}-3\frac{\Delta h_{+}+\Delta h_{-}}{{\left(h_{+}-h_{-}\right)}^{3}}h_{+}h_{-}.$$

The transverse component of velocity, *v*, is obtained from (8) by integrating Eq. (3) subject to (4), giving

$$v=U[-\frac{A_{x}}{3}(y^{3}-h_{-}^{3})-\frac{B_{x}}{2}(y^{2}-h_{-}^{2})-C_{x}(y-h_{-})],$$ (12)

where $A_{x}=\partial A/\partial x$, etc. Finally, it may be verified by differentiation that the streamfunction is

$$\psi =U[-\frac{h_{-}}{2}+\frac{A}{3}(y^{3}-h_{-}^{3})+\frac{B}{2}(y^{2}-h_{-}^{2})+C(y-h_{-})],$$ (13)

where the fluid velocity components are given by u=∂ψ/∂y and v=−∂ψ/∂x.

**FIG. 1.**
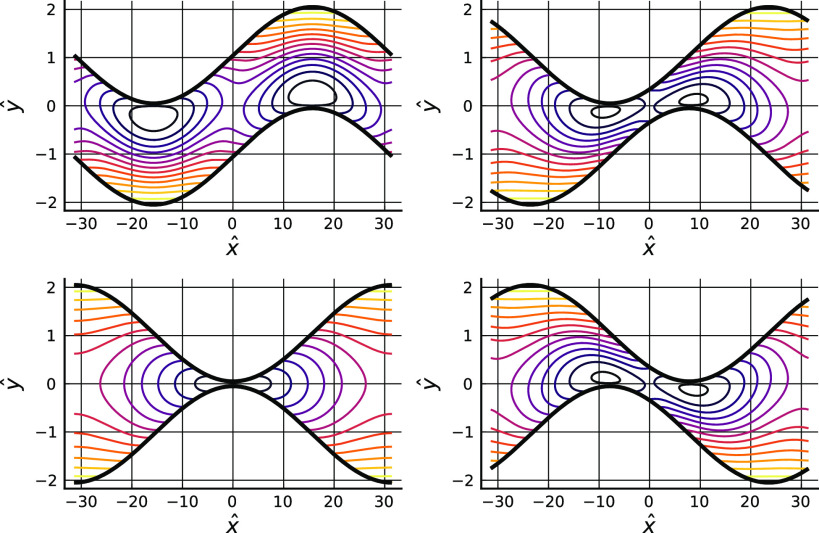
The streamfunction *ψ* at t=2π(0,0.25,0.5,0.75)/kU (plots ordered left to right and top to bottom). Parameter values: η=0.1, ka=0.1.

Figure 1 illustrates instantaneous streamlines at a sequence of times spanning half a period, as seen in the laboratory frame in which the two solid boundaries are moving in opposite directions. Fluid moves with the boundaries in the regions near the maxima of the upper boundary and the minima of the lower boundary, and in the band on either side of the center of the gap fluid is pushed away from the forward moving or leading edges of the sinusoids, and sucked in toward the trailing edges. At some times, but not all, there are front and rear stagnation points on the boundaries, which shift position along the boundaries as these move relative to one another.

**FIG. 2.**
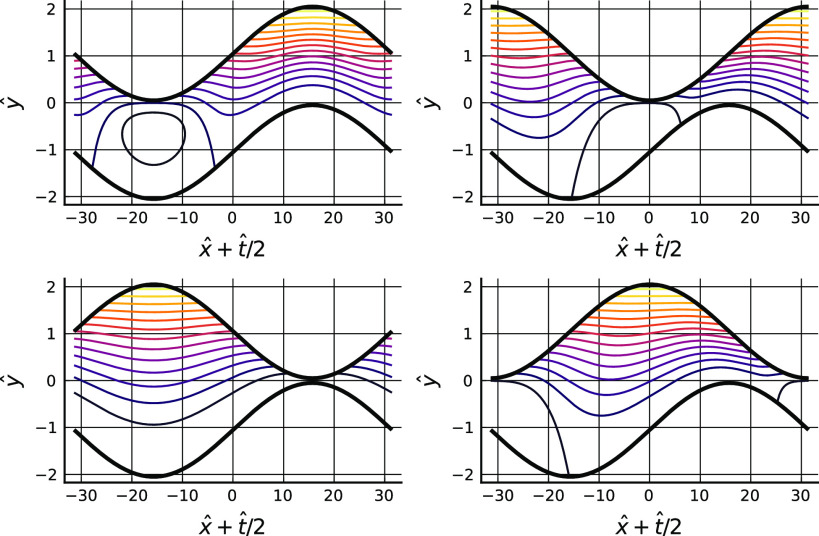
The streamfunction in a frame of reference moving with the lower wall, ψ+(Uy/2), at t=2π(0,0.25,0.5,0.75)/kU. Parameters as in Fig. 1.

Figure 2 shows streamlines at the same times but in a frame of reference moving with the lower boundary. The density of streamlines is low in the part of the gap near the lower boundary, which indicate that fluid fluxes are low there, i.e., the fluid is mainly being carried along with the wall. However, streamlines linking points on the lower boundary are visible at some times, but not all, which indicates that there are sometimes locations where material can more easily detach from, or conversely be deposited onto, the lower wall of the gap.

**FIG. 3.**
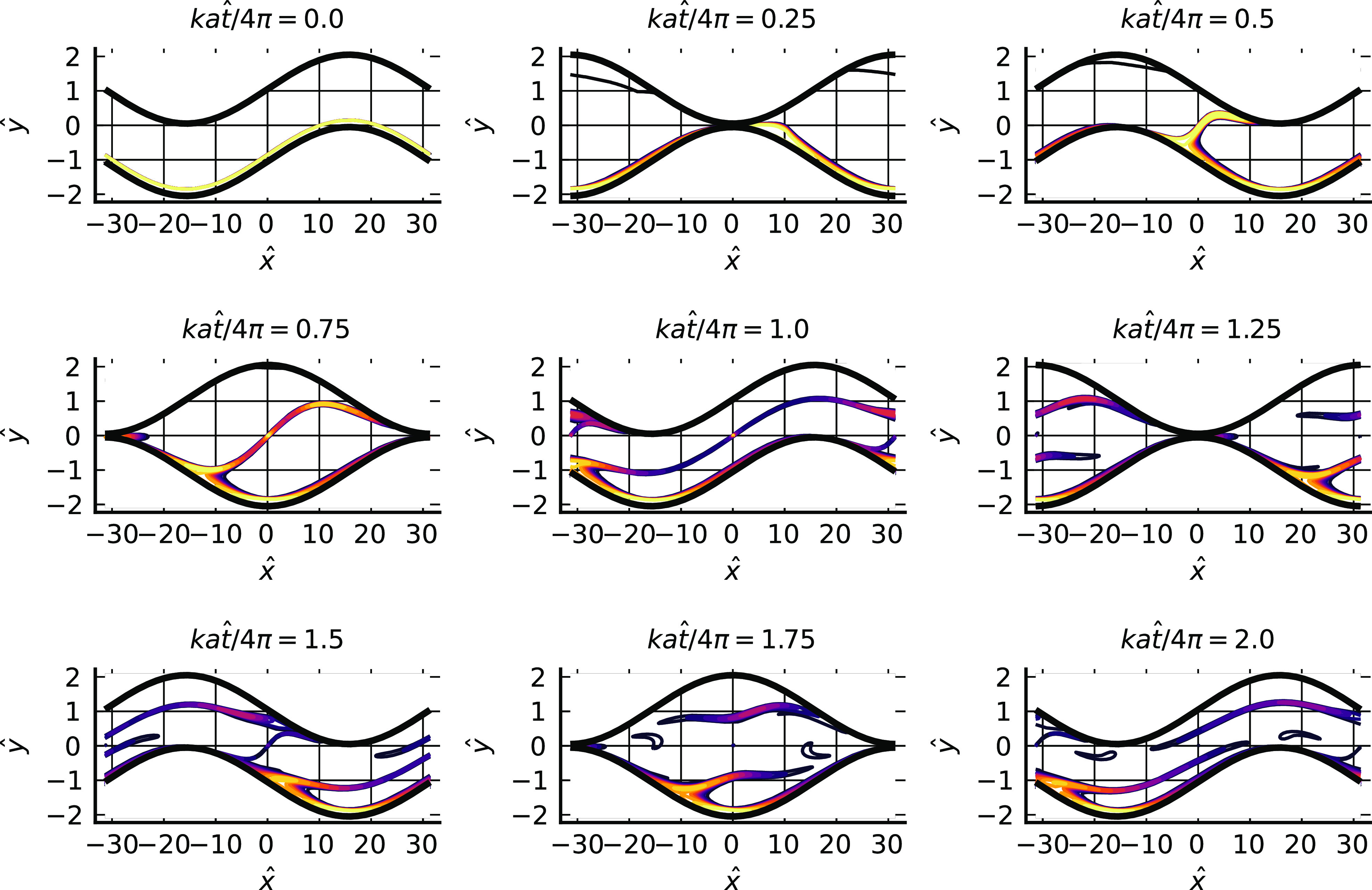
Concentration of a non-diffusing tracer at times t=4π(0,0.25,0.5,0.75,1,1.25,1.5,1.75,2)/kU. Parameters as in Fig. 1. The concentration is initially zero everywhere except in a strip of width 0.2a adjacent to the lower boundary.

Figure 3 further visualizes the flow field by tracking the concentration of a non-diffusing passive tracer initially present only in a finite width strip against the lower boundary. The 2D tracer advection equation is solved using dimensional splitting on a 512 × 1024 regular grid, with each 1D sweep solved using a second order MUSCL-Hancock scheme; see Toro,^18^ Chaps. 13 and 16. The fluid velocity is taken to be (u,v)=(±U/2,0) when *y* lies outside the interval $\left[h_{-}(x,t),h_{+}(x,t)\right]$. The results have relevance to the particle tracking calculations of the rest of this document since Péclet numbers there are large, but care should be taken in interpreting these results for this purpose since the width of the initial tracer concentration strip in the computations is relatively large compared to the range of attraction of the particle trapping force.

From these plots of concentration in the laboratory frame, it can be seen that the tracer is spread along and across the gap by repeated creation of filaments, which originate at the points of close approach of the two surfaces and are subsequently stretched along and transported across the channel. Each close approach event generates a new filament, which is further elongated in the axial direction during each subsequent period, thus distributing the tracer into an ever increasing set of thin layers, and hence generating large transverse concentration gradients which in turn will generate large transverse diffusive fluxes, so bringing about effective cross-gap transport of tracer. At the same time, some tracer is contacted immediately with the other wall of the channel at the instants of close approach, provided the initial layer thickness is comparable to the minimum spacing between surfaces. Also, slow movement of tracer takes place within the layer on the lower wall, as is evidenced by variations in thickness and concentration of the layer at points away from the highest point of the lower wall. These observations suggest that even at zero Reynolds number in a comparatively simple geometry complicated Lagrangian trajectories can result, with different time scales depending on whether processes occur near a wall or in the bulk of the fluid. The motion of particles convected in the flow may therefore have surprising complexity. In the context of mass transport, when the lower surface represents a bar of soap and the upper surface a dirty hand, the filiation process described above could be an important mechanism generating a good rate of surfactant transport from source to the place where it is needed, despite the overall high Péclet number.

In the symmetrical geometry assumed here $h_{-}(0,t)=-h_{+}(0,t)$, and so $\Delta h_{+}+\Delta h_{-}=h_{+}+h_{-}$, and thus G(0,t)=0. Furthermore, the assumed symmetry implies that *G* is an odd function of *x*, which for spatially periodic *G* implies that its integral over a period is zero. This in turn implies that the pressure *p*(*x*, *t*) is periodic in *x*. So in a finite length geometry the pressure can be thought of as the sum of a zero mean periodic component, forced by the local geometry, and a constant value set by matching to conditions at the ends of the gap. As argued in Sec. IV H of Batchelor,^16^ this constant pressure can be large, $O(\mu U/ka^{2})$, a factor of 1/ka larger than the wall shear stresses, see Appendix for an explicit calculation. It follows that if the two surfaces are pushed together by an external force per unit area P, then, assuming this force is balanced by lubrication pressure alone and solid–solid contacts play no part, the order of magnitude of the thickness of the fluid-filled gap must be $a∼\sqrt{\mu U/kP}$. Taking P to be of order a few times $10^{2}\;{N/m}^{2}$ in hand washing, and $2\pi /k∼10^{-3}\;m$, with other parameters as above, then $\sqrt{\mu U/kP}∼10^{-5}\;m$ justifying the value of *a* assumed above. Furthermore, $ka∼10^{-1}$ which is indeed small.

## IV. PARTICLE MOTION

We consider a particle of radius *d*, with instantaneous position X(t), which interacts with the wall through a force given by the gradient of potential ϕ. The total force on the particle must vanish, and so, by virtue of the linearity of the Stokes equations and boundary conditions,

$$0=-\nabla \phi +R_{X}^{f}[u]-R_{X}^{p}\cdot \dot{X},$$ (14)

where $R_{X}^{f}$ is the linear functional linking the force on a stationary particle at X to the fluid velocity field, and $R_{X}^{p}$ is the resistance tensor for a particle at X moving through stagnant fluid, in both cases in the presence of the channel boundaries Cox.^19^ Consistent with the small slope assumption underlying the use of lubrication theory, the particle-wall interaction potential is taken to be

$$\phi =\beta [{\left(\frac{l}{y-h_{-}}\right)}^{m}-{\left(\frac{l}{y-h_{-}}\right)}^{n}],$$ (15)

with *l* the characteristic distance from the wall over which the potential is significant. Then, assuming $R^{f}$ and $R^{p}$ to be isotropic and to take Stokes' law form for a particle of radius *d*, i.e., that the particle is small not too close to the wall,

$$\dot{X}=u-\frac{1}{6\pi d\mu }\nabla \phi ,$$ (16)

where the fluid velocity and potential gradient are evaluated at (X,t). This equation is easily stepped forward in time numerically so as to find particle trajectories.

The treatment of the hydrodynamic force on the particle in Eq. (16) is approximate. In particular, no account is taken of the consequences of proximity to the bounding wall on the forces on the particle.^20^ This is an acceptable approximation when the distance of the particle from the wall is large compared with *d*, but is questionable when the particle is close to the wall. Broadly speaking wall effects create an additional resistance to particle motion, and so cause particles to lag behind the local fluid flow when the distance of the particle from the wall is not large compared to its diameter. Hence, expressions which take no account of the presence of nearby walls are likely to overestimate particle velocities, and in the present context this is likely to translate into an overestimate of the ease with which particles can be removed from the near-wall region. In principle, it is possible to include finite particle size wall interactions in the calculation using the results of the paper of Cox cited above,^21^ but in practice the significant additional complication seems likely only to cause quantitative changes rather than induce new qualitative phenomena.

Inertial lift is also neglected, but since as argued in Sec. II the particle Reynolds numbers are small, lift will be a much smaller force than drag and inconsequential except perhaps on long time scales. However, there is a further subtlety in the present context since it is the drag associated with wall-normal velocity components, rather than wall-parallel, which drives particle removal, and so inertial lift forces should be compared with drag associated with wall-normal motions. Since in the lubrication approximation wall-normal velocities are smaller than wall-parallel by O(ka), neglect of lift requires that particle Reynolds numbers be small compared to *ka* rather than to 1, which is indeed the case for the parameter values assumed here. See Magnaudet *et al.*^22^ and Ekanayake *et al.*^23^ for recent studies of the hydrodynamic forces on a particle near a wall with particle motion or undisturbed flow in the parallel direction and including inertial effects, and Maxey and Riley^24^ for a discussion of (small) contributions from unsteadiness and velocity profile curvature to the force on a particle in unbounded fluid.

The interaction between virus particle and surface must in reality be far more complex than that represented by Eq. (15). Extremely detailed information at the molecular level is now becoming available,^25^ but here a lumped description at the continuum scale is required. Fortunately, recent advances in mesoscopic simulation^26,27^ hold out the possibility of elucidating the various modes and processes of virus-surface interaction, and estimation of force, range, and mobility parameters at the appropriate scale.

It follows from Eq. (16), using (15), that the dimensionless parameter

$$B=\frac{\beta }{6\pi d\mu \text{Ul}}$$ (17)

measures the relative strength of wall attraction to hydrodynamic forces when velocity differences between particle and fluid are of order *U*. For convenience, we introduce λ=l/a and δ=d/a so that $B=\beta /(6\pi a^{2}\mu U\delta \lambda )$. The small parameters *λ* and *δ* are likely to be comparable in magnitude, with λ>δ.

Since axial velocities at a distance O(l) from the wall are expected to be of order Ul/a=λU in a frame of reference moving with the wall (when $h_{+}-h_{-}=O(a)$ and $\partial u/\partial y{|}_{\text{wall}}\neq 0$), and in lubrication theory transverse velocities are smaller than axial by a factor of wall slope ka, we might infer from Eq. (16) that particles cannot be pulled out from the trap by transverse flow if B exceeds O(kaλ). We shall find below that this criterion over-estimates the strength of hydrodynamic forces and weaker potentials can in fact hold particles trapped; the underlying cause is the significant time and space variations of the fluid velocity.

## V. RESULTS

**FIG. 4.**
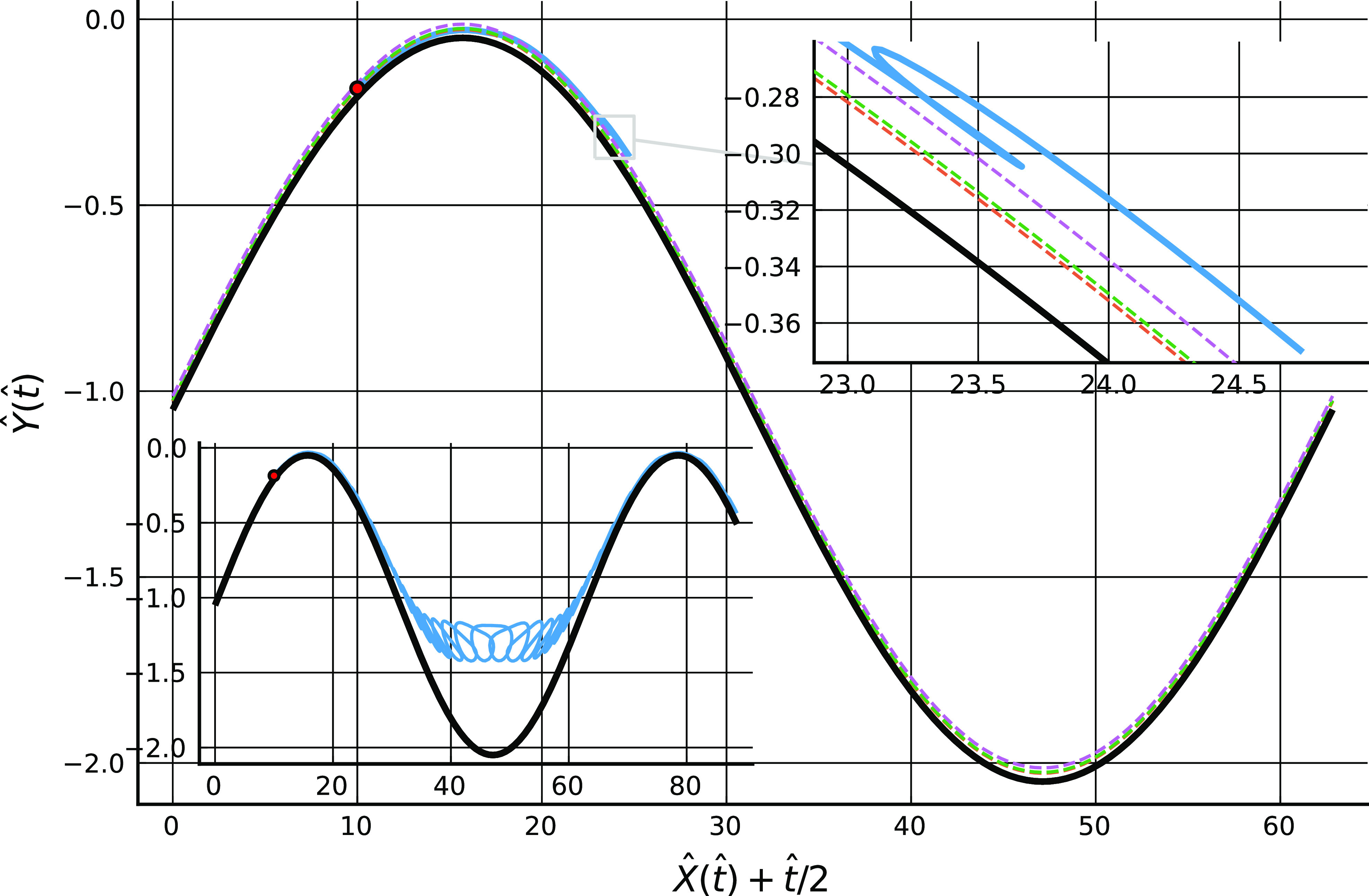
In the main figure, an example particle trajectory, X(t) (blue line, starting from the red dot at *t* = 0), plotted in a frame of reference moving with the lower wall for 0≤t≤3(2π/kU), with $B=10^{-7},\;ka=0.1,\;\lambda =0.02$, *m* = 12, *n* = 6, and other parameters as Fig. 1. The black line is the channel lower wall, and the three dashed lines are the locations of the potential minimum, the point of maximum attractive force, and the outer edge of the potential. The particle travels in the direction of positive *x* to the point of escape form the wall attraction. In the two insets, a close-up of the trajectory at escape, and an extension of the calculation to t=30(2π/kU).

**FIG. 5.**
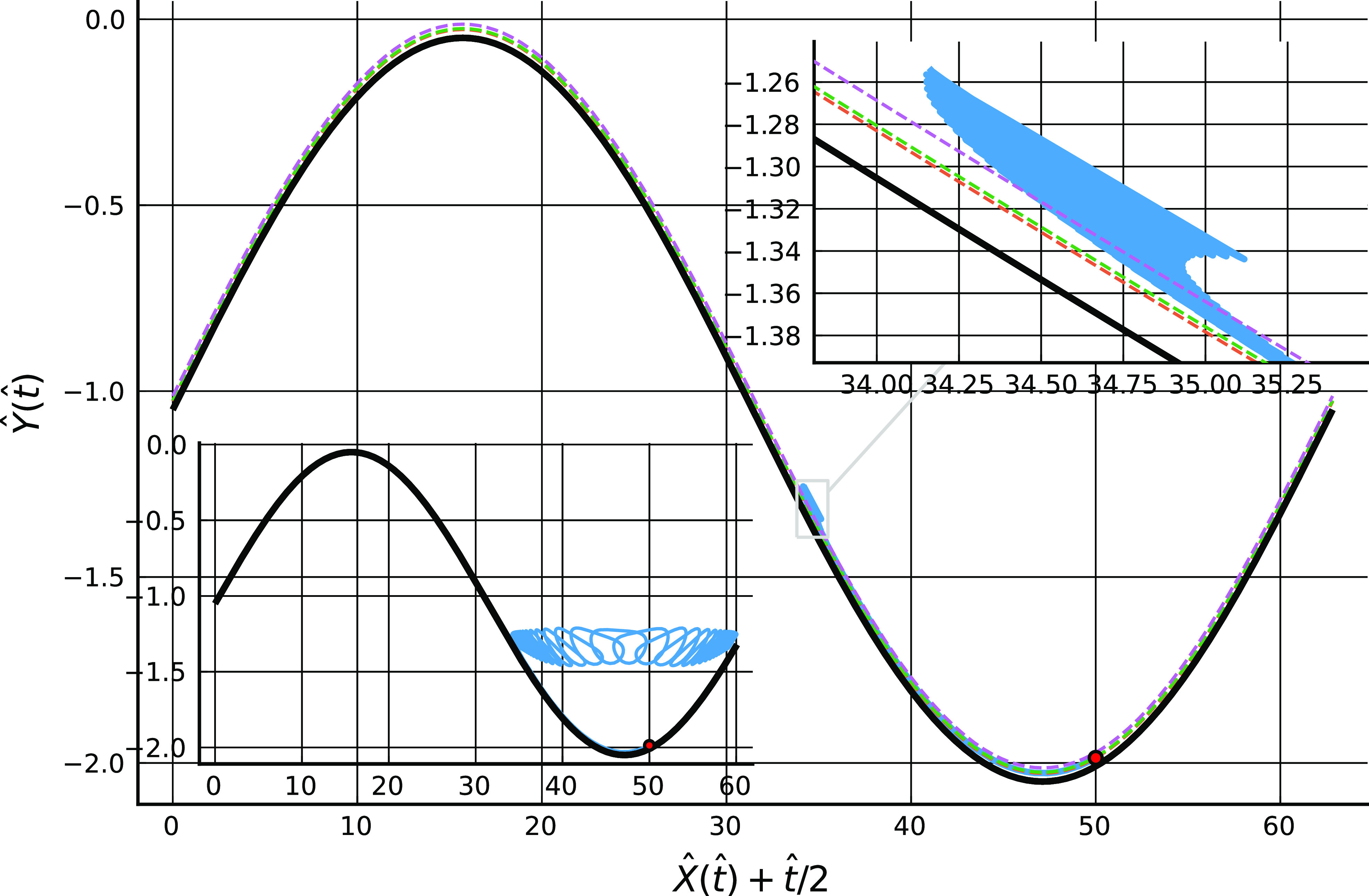
Particle trajectory, X(t) (blue line, starting from the red dot at *t* = 0), plotted in a frame of reference moving with the lower wall for 0≤t≤120(2π/kU) with parameters as Fig. 4. The initial location of the particle is such that it travels toward negative *x* to the point of release. In the lower inset, the calculation is extended to t=160(2π/kU).

Figure 4 shows a computed trajectory with ka=η=0.1 and $B=10^{-7}$ where the particle travels to the right, in a frame of reference moving with the lower wall, from its initial position on the lower wall to the point of escape from the trapping potential. A further calculation, not illustrated here, indicates that the particle remains trapped near the wall when starting from this position if $B=2\times 10^{-5}$. Figure 5 shows a trajectory where the particle travels to the left from its starting position on the lower wall to the point of escape, again with ka=η=0.1 and $B=10^{-7}$.

In both cases, the particle moves far from the wall and into bulk fluid once it has escaped from the potential well, although after sufficiently long times it is re-trapped (and presumably eventually re-released since the flow field is periodic in time, so a similar release-trap scenario will play out over and over again).

The locations of release from the trap differ in the two figures. In Fig. 4, the particle becomes free, in the sense of moving to a position where the magnitude of the potential has fallen to one tenth of its value at the minimum, at a point just downstream (i.e., to the right) of the peak of the wall profile at X+Ut/2≈17, whereas in Fig. 5 the release occurs near the midpoint between the maximum and minimum of the wall profile at X+Ut/2≈35. In the first case, release seems to be associated with the strong shearing flow occurring when the wall profiles coincide in such a way as to give a narrow channel gap, while in the second case the release process appears to be associated with weaker flows in the sheltered region in the lee of the roughness element. Other simulations, not reported here, indicate that release only occurs at one of these two positions and in these ways. From examination of a variety of results, it appears that particle release involves first transport along the wall to a position where conditions in the vicinity are favorable for release to occur, and then motion with a component normal to the wall driven by the local flow field, which may occur over many periods, during which the particle is transported to and beyond the edge of the potential well.

**FIG. 6.**
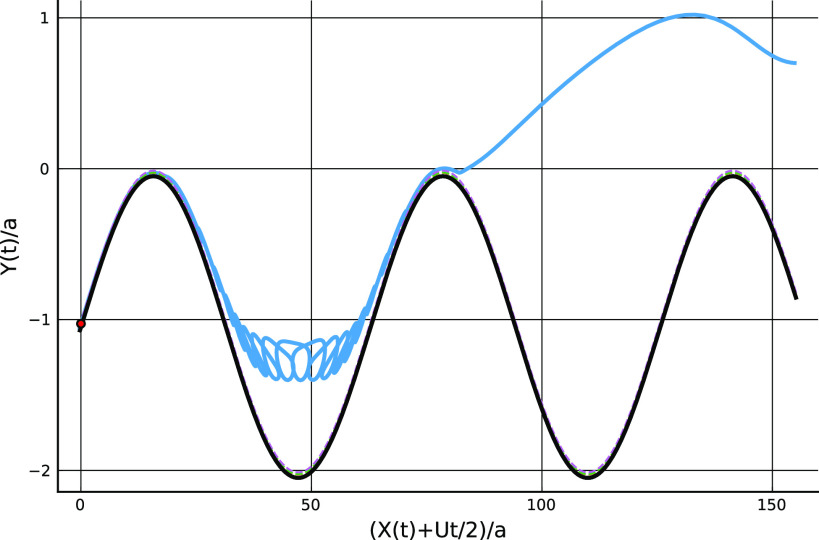
A case where the particle crosses to the other side of the channel, 0≤t≤45(2π/kU). $B=10^{-6}$ and other parameters are as in Fig. 4.

The particle is not constrained always to move in y<0. Parameter values and starting point can be chosen so that the trajectory crosses the channel centerline, as illustrated in Fig. 6, although such cases seem difficult to find. Presumably it is important for the particle to arrive at the top of the roughness at just the right moment.

**FIG. 7.**
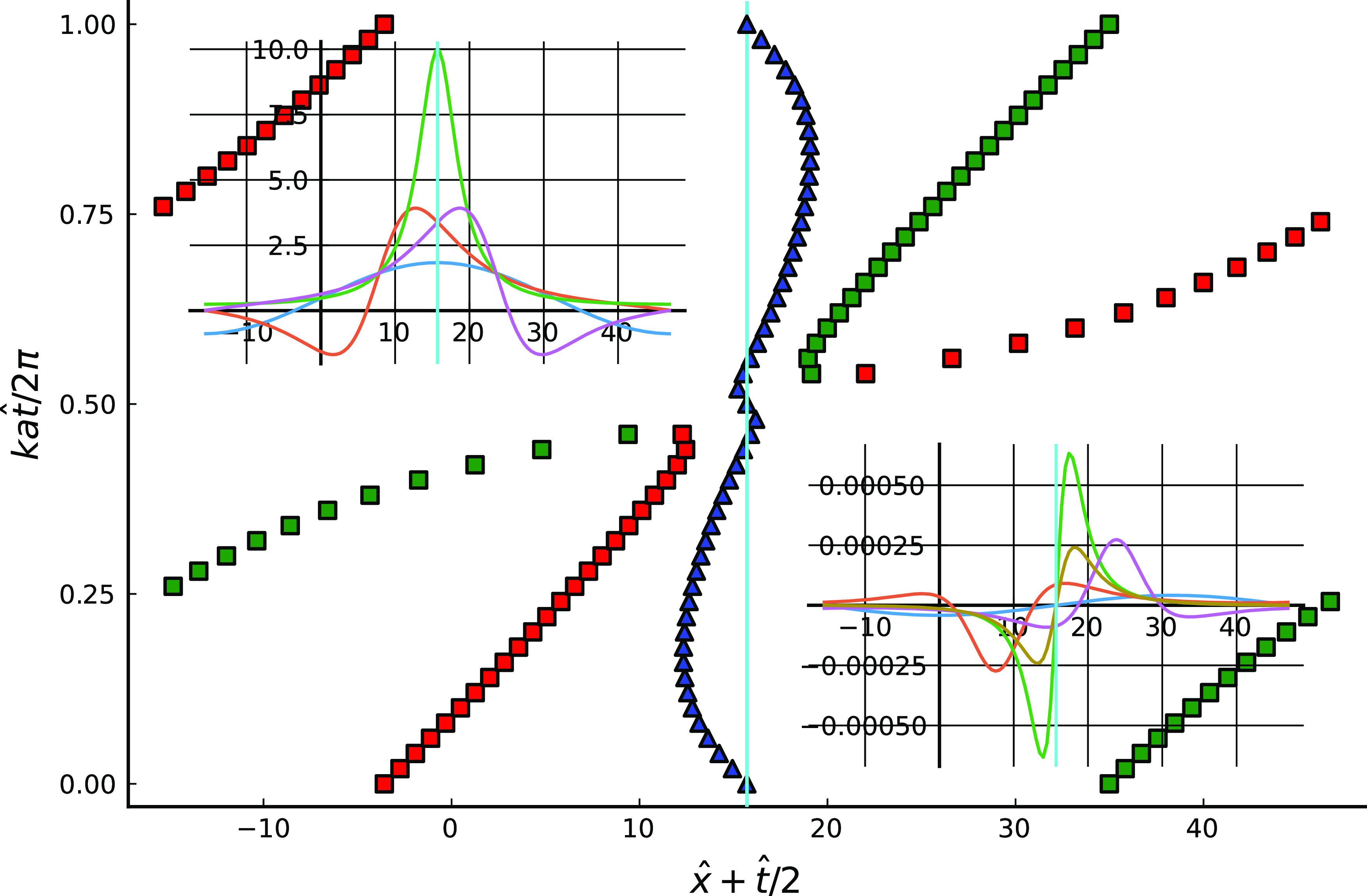
In the main plot, a map of special points of $u(x,h_{-},t)$, for ka=η=0.1. Blue triangles indicate the point of maximum positive wall shear stress; squares points, where $\partial u/\partial y(x,h_{-},t)=0$, red for positive $\partial^{2}u/\partial x\partial y(x,h_{-},t)$, green negative; the vertical cyan line marks the position of the maximum of $h_{-}$ throughout. In the upper left subplot, $\partial \hat{u}/\partial \hat{y}$ at t=2π(0,0.25,0.5,0.75)/kU (blue, red, green, and mauve). In the lower right subplot, ${\hat{u}}_{n}(h_{-}+l,t)$ at the same instants, with the additional light brown line showing the time average $\bar{{\hat{u}}_{n}}$.

In order to help identify the processes responsible for particle untrapping, Fig. 7 maps out the positions of various significant wall shear stress values, in the frame of reference moving with the lower boundary. The triangles in Fig. 7 give the location of the maximum, and the upper left subplot shows the values, of the shear stress on the lower wall at various times. It can be seen that the shear stress maximum is located near the maximum of $h_{-}$ and takes its greatest value when the tips of the upper and lower boundary roughness elements are directly opposite one another at kUt/2π=0.5. The squares in Fig. 7 show the time-varying positions of points of zero wall shear stress, which are significant in the present context since they are associated with stagnation points. Green squares show positions where $\partial^{2}u/\partial x\partial y(x,h_{-},t)<0$, and red squares where this derivative is positive. At a small distance above the lower wall fluid flows toward the green points and away from the red points, and there are corresponding outward flows in the vicinity of the green points and inward flows in the vicinity of the red. The lower right subplot shows the values at various times of the wall-normal velocity at a position close to the potential minimum and its time average. Positive time average wall normal velocities are only found to the right of the maximum of $h_{-}$ and are mainly generated at times between kUt/2π=0.5 and kUt/2π=1. To summarize, the largest instantaneous positive value of normal velocity is found just downstream of the maximum of $h_{-}$, while positive time-average normal velocity is found to the right of the maximum. Hence, fast particle escape processes are likely to occur near the maximum, and slow escape processes playing out over many periods will occur to its right. At any instant particles will move parallel to the wall away from the red squares, and toward the green, so sweeping them toward the maximum of $h_{-}$ if they start a short distance to its left, or sweeping them into the region behind the maximum if they start to its right or far to its left.

It is interesting to compare the critical values of B for release to occur, either from near the “top of the roughness,” as in Fig. 4, $B_{\text{crit}}(\text{top})$, or from a “rear stagnation point,” as in Fig. 5, $B_{\text{crit}}(\text{rear})$, see Table I, where values of *λka* and some other scaling groups are given for purposes of comparison. Both critical values indeed scale with *λka* over the range examined but it is clear that in both cases the numerical coefficient linking *λka* and $B_{\text{crit}}$ is small, which suggests that the argument of Sec. IV needs refinement. The critical value for detachment is much larger when the process occurs at the top of the roughness than on the rear flank, because hydrodynamic forces are significantly amplified at the peak when the gap between the surfaces is at its narrowest and so are able to overcome the attraction of stronger potentials.

**TABLE I.** Ranges containing $B_{\text{crit}}$ as *ka* is varied, with λ=0.02, η=0.1, *m* = 12, and *n* = 6.

*ka*	$B_{\text{crit}}(\text{top})$	$B_{\text{crit}}(\text{rear})$	kaλ	$ka\lambda^{2}/20\eta$	$ka\lambda^{2}/100$
0.2	$(3.5,4.0)\times 10^{-5}$	$(6.0,6.5)\times 10^{-7}$	$4\times 10^{-3}$	$4\times 10^{-5}$	$8\times 10^{-7}$
0.1	$(1.75,2.0)\times 10^{-5}$	$(2.75,3.0)\times 10^{-7}$	$2\times 10^{-3}$	$2\times 10^{-5}$	$4\times 10^{-7}$
0.01	$(1.75,1.9)\times 10^{-6}$	$(2.875,3.0)\times 10^{-8}$	$2\times 10^{-4}$	$2\times 10^{-6}$	$4\times 10^{-8}$
0.001	$(1.5,2.0)\times 10^{-7}$	$(3.0,3.1)\times 10^{-9}$	$2\times 10^{-5}$	$2\times 10^{-7}$	$4\times 10^{-9}$

The scaling argument given in Sec. IV can now be refined, using ideas gleaned from the simulations. The first aspect to address is that it is the velocity component normal to the duct wall in a frame of reference moving with the wall, $u_{n}=v-(u+\frac{U}{2})\partial h_{-}/\partial x$, which is important for particle removal from the trap, rather than the transverse component *v*, the main contribution of which is simply to cause particles to terrain-follow the wall. General considerations based on ∇·u=0 and the no-slip boundary condition lead to the estimate $u_{n}=O({\left(\partial^{2}u_{t}/\partial t\partial n\right)}_{y=h_{-}}{\left(y-h_{-}\right)}^{2})$ at most, where *u_t_* is the tangential component of velocity (equal to leading order in lubrication theory to the axial component, *u*) and the partial derivatives are in the tangential and normal directions. Hence, the normal velocity at a distance O(l) from the wall is $O(\lambda^{2}a^{2}\gamma_{x})$, where *γ_x_* is the axial rate of change of the shear rate at the wall. Explicit calculation using Eq. (8) shows that *γ* receives a contribution $O(U/(h_{+}-h_{-}))$ due to the local shearing motion of the channel walls, together with a contribution $O(U(\Delta h_{+}+\Delta h_{-})/{\left(h_{+}-h_{-}\right)}^{2})$ from the pressure gradient driven flow. Away from the narrowest point of the gap, these contributions are comparable and lead to $\gamma_{x}=O(kU/a)$, and thence $u_{n}=O(\mathrm{kaU}\lambda^{2})$. On the other hand, near the narrowest point of the gap, when *η* is small, it might seem that the pressure gradient contribution to *γ* dominates since it scales with the inverse square of the gap width; however, this is misleading since with the present highly symmetrical geometry its numerator vanishes when the two surfaces are exactly aligned and is small at times close to that instant, and so the correct estimate is $\gamma_{x}=O(kU/\eta a)$, whence $u_{n}=O(\mathrm{kaU}\lambda^{2}/\eta )$.

The simulations show that untrapping near the top of the roughness occurs within a single period 2π/kU, which requires that the particle normal velocity to be at least O(kUl/2π). This is not in contradiction with the estimate given at the end of the previous paragraph for *u_n_* provided 2πλ/η=O(1), and so untrapping by this means it requires distances of approach between the surfaces comparable to the range of the potential well.

For particle detachment to occur, the hydrodynamic drag forces on the particle must exceed the wall attraction force, and so $6\pi d\mu v_{\text{slip}}\geq O(\beta /l)$, with $0<v_{\text{slip}}<u_{n}$, the lower velocity bound arising because some slip is needed for a drag force to be generated, and the upper because positive particle velocity is required for the particle to move outward. Since little time is available for top detachment in a single encounter between roughness peaks, it seems reasonable that $v_{\text{slip}}$ can only be a small fraction of *u_n_*, say $v_{\text{slip}}=\alpha u_{n}$ with *α* numerically small but not so small that inadequate drag forces will be generated. Combining with the previous estimates we estimate $B_{\text{crit}}(\text{top})=O(\alpha ka\lambda^{2}/\eta )$, and picking α=1/20 gives good agreement in Table I.

In contrast, the simulations indicate that detachment at the rear is a gradual process taking place over many periods. The particle oscillates along the tangential direction once it has drifted to the vicinity of the release point and moves outward under the action of a small but non-zero time-averaged component in *u_n_*. There is no constraint that the particle's normal velocity be large enough to complete removal in a finite number of periods; it just has to be positive on average. This is difficult to analyze because the order of magnitude estimates made so far relate to typical values of *u_n_*, and since *u_n_* is observed in the simulations to take both positive and negative values its time average may be very different, and potentially much smaller, than its typical value. Since the two surfaces are in steady shear relative to one another the time average of *u_n_* can be non-zero and had the surfaces been in oscillation, causing the flow to periodically reverse, a zero time-average would be expected since Stokes flows are reversible. In the absence of a quantitative estimate, we assume that the time average of *u_n_*, denoted $\bar{u_{n}}$, is equal to some small fraction *ε* of the typical value $\bar{u_{n}}=O(\varepsilon \mathrm{kaU}\lambda^{2})$. On this basis, assuming $v_{\text{slip}}\approx \bar{u_{n}}$, and then balancing time-average drag against attraction, we obtain $B_{\text{crit}}(\text{rear})=O(\varepsilon ka\lambda^{2})$. Taking $\varepsilon =10^{-2}$ matches the observed values of $B_{\text{crit}}(\text{rear})$, see Table I. The dimensional time for a particle to move a distance of order *l* away from the wall is $l/\bar{u_{n}}=O(a/\varepsilon \text{ka}\lambda U)$, which, for $\varepsilon =10^{-2}$ and the parameter values given in Sec. II is 20 s, which is of the same order of magnitude as the hand washing time recommended by the WHO.^6^

## VI. CONCLUSIONS AND REMARKS

Particle tracking calculations and solutions of the tracer advection equation in the time-varying gap between relatively moving corrugated surfaces show that surprisingly complicated particle trajectories and concentration distributions can arise in a comparatively simple geometry at low Reynolds number. Tracer layers near a wall can be pulled out into a series of thinning layers through processes occurring at the points of close approach between the surfaces, and the resulting large transverse concentration gradients may drive significant cross-gap diffusive transport even though bulk Péclet numbers are large. This mechanism may be important for effective delivery of soap from a bar to the surface of dirty hands.

Bound particles are removed from all points on the corrugated surface provided B is less than a critical value, $B_{\text{crit}}$. The scalings of Table I imply that ${\left(\beta a/6\pi d\mu Ul^{3}k\right)}_{\text{crit}}∼1$. So narrower gaps (i.e., smaller *a*), steeper roughness (larger *k*), faster relative movement of surfaces or larger fluid viscosity, all mean that particles can be dragged off the wall despite larger values of *β*. Larger particles (i.e., larger values of *d*, or larger values of *l*) are also easier to remove in this sense. In all cases, the key effect is a strengthening of hydrodynamic drag forces, either directly, e.g., via the *U*, *d* and *μ* dependences in Stokes' law, or indirectly through the increase in normal velocity with distance from the wall.

The timescale for particle removal through the gradual process acting away from points of close approach of the surfaces is O(a/εkaλU), and for the parameter values chosen here this turns out to be rather close to the time recommended empirically to be spent in routine hand washing. The numerical closeness is fortuitous, since it is dependent on the precise values of film thickness and surface roughness parameters chosen, but the order of magnitude correspondence is suggestive that the hydrodynamic theory developed here has captured something of the essentials of the process.

If B is not large, then particles are not removed but rather are swept to accumulate at a point on the downstream side of each roughness element (a “rear stagnation point”).

With the present model of particle-surface interaction, particles roll or slide along in the potential well until they possibly reach a position where hydrodynamic forces are strong enough to pull them out of the trap. If the interaction sites on the wall had been represented as a set of discrete points, we might expect the particle either to be bound to a fixed site if B were large, or at smaller values to hop along the wall from site to site under the action of tangential hydrodynamic forces until reaching a point were hydrodynamic forces acting normal to the wall were strong enough to free it completely and then transport it a significant distance from the wall. A shared feature of the simulation results reported here is that transport along the wall is a faster process than transport normal to the wall, reflecting the character of the near-wall velocity components. We might expect similar behavior to be observed in a discrete binding center model, and so again particle transport to positions far from the wall will occur only at special points on the surface.

As a crude summary conclusion, it seems fair to say that particle transport into the bulk is not an easy process since it requires a threshold flow strength to be exceeded, requires particles to be transported along the surface to certain discrete points where the flow conditions are particularly favorable for particle untrapping to take place, and for most starting positions, it requires a time extending over many periods of motion of the periodic surfaces for completion.

The consequences of chemical attack on a virus particle by active ingredients introduced during the washing process have not been considered here. Several possibilities deserve study: the trapping attraction between the particle and the surface might be weakened, and the rate of transport of an active ingredient to the surface will set the timescale over which this process may be important; the particle may be deactivated without leaving the surface, and again transport rates from bulk to surface must play a key role; finally, chemical deactivation of a particle could occur within the bulk fluid after the particle has been detached from the wall, which involves an interplay between the time scales for release from trapping, for transport of particles away from the wall, and for transport of active ingredients toward the wall. The clothes laundry literature is clearly of relevance.^28^

The present model implies that there if wall force attractions are too strong, i.e., if B is too large, then particles are not removed from wall into the bulk fluid and remain trapped. Although in practice this hard threshold will be smeared by a distribution of roughness geometry parameters or trap strengths, we might conjecture that deactivation of particles by action of surfactant whilst they are still bound to the wall will not display threshold effects in *U*, because there is no threshold in the advection-diffusion equation for surfactant transport other than that arising from effects associated with total time of exposure, whereas deactivation occurring after particles has been detached into the bulk will show a *U* threshold. This may be a way of telling the two possibilities apart.

Throughout, the geometrical parameters *a* (average channel width) and *ηa* (channel width at points of closest approach of roughness elements) have been taken as given, but they can seem to influence the critical value of wall attraction strength *β* in the scalings found in Table I. In reality, we might expect both of these length scales to be determined dynamically through an interaction between the applied normal load, the lubrication pressure developed in the gap as the surfaces slide relative to one another, and potentially also the deformability of the bounding surfaces. Calculation of the pressure within the gap, by integration of Eq. (11), requires specification of how the surfaces diverge from one another as x→±∞. Once this is done and the pressure is found, it can be integrated to give the normal force on each surface, and then the time-averaged result equated to the applied load so as to obtain an equation determining *a*.

Incorporation into the analysis of elastohydrodynamic effects^29,30^ would be an interesting extension of this calculation, since deformations are likely to occur at the narrowest points of the gap so changing the geometry and flow field in a region where particle detachment occurs. The author's elastohydrodynamic calculations, which it is hoped will be reported in a future publication, indicate that surface deformations flatten the channel walls and thus reduce the magnitude of flows acting to lift particles from the surface; all other factors held constant, and it is therefore harder to remove a particle from a deformable wall.

Experimental inputs, on roughness characteristics, gap widths, and surface compliances, as well as on particle-surface interaction forces, are all vitally important if any of these ideas are to be tested through quantitative predictions. A more sophisticated representation of surface roughness^31^ is also desirable, as is extension to geometries and flows in three dimensions.

Finally, the model used here for fluid forces on a suspended particle near a rigid wall is crude and should be improved to include the consequences of wall proximity, and perhaps also fluid inertia and particle Brownian motion.

Use of lubrication theory has allowed a first examination of an interesting and practically relevant phenomenon, through nothing more difficult than numerical integration of a small ODE system on a laptop computer. It seems to the author that this time-honored approximation can still be put to good use.

## AUTHORS' CONTRIBUTIONS

P.S.H. did everything reported here.

## Data Availability

The data that support the findings of this study are available within the article.

## APPENDIX: LOAD CARRYING PRESSURE

For symmetrical, but not necessarily periodic, functions $h_{\pm }$ satisfying $h_{-}(-x,t)=-h_{+}(x,t)$, the pressure within the fluid-filled gap is found from Eq. (11) as

$$p(x,t)-p_{0}=6\mu U\int_{x}^{\infty }\frac{h_{+}+h_{-}}{{\left(h_{+}-h_{-}\right)}^{3}}dx\prime ,$$ (A1)

where *p*_0_ is the pressure in the surrounding fluid within which the moving surfaces are immersed. Suppose

$$h_{+}(x,t)=a(1+\frac{\eta }{2})+a\{\begin{array}{cc} {\left[k(x-L-\frac{Ut}{2})\right]}^{q}, & x\geq L+\frac{Ut}{2}\\ 0, & x<L+\frac{Ut}{2}. \end{array}$$ (A2)

Then for $|x|<L+\frac{Ut}{2}$,

$$\begin{array}{c} p(x,t)-p_{0}=\frac{6\mu U}{a^{2}}\int_{0}^{\infty }\frac{{\left(kx^{\prime }\right)}^{q}}{{\left((2+\eta )+{\left(kx^{\prime }\right)}^{q}\right)}^{3}}dx\prime \\ =\frac{6\mu U}{a}\frac{1}{ka}\frac{g(q)}{{\left(2+\eta \right)}^{3/2}}, \end{array}$$ (A3)

where

$$g(q)=\int_{0}^{\infty }\frac{x^{q}}{{\left(1+x^{q}\right)}^{3}}dx.$$ (A4)

This integral is given in Gradshteyn and Ryzhik;^32^ for example, g(2)=π/16 and so when η≪1 the numerical coefficient in Eq. (A3) is $6\pi /2^{11/2}\approx 0.42$.
